# Improving the Dosing Schedules of Targeted Anticancer Agents

**DOI:** 10.3390/ph18060848

**Published:** 2025-06-06

**Authors:** Dominique Levêque

**Affiliations:** Pharmacy, Hôpital de Hautepierre and Pharmacy, Institut de Cancérologie Strasbourg Europe (ICANS), Avenue Molière, 67000 Strasbourg, France; dominique.leveque@chru-strasbourg.fr; Tel.: +33-388-128-213

**Keywords:** targeted therapies, cancer, dose, safety, dosing schedule

## Abstract

Beyond developing new agents, cancer treatment can also be optimized by modifying the dosing regimen of approved drugs. Academic teams have experimented with different ways of improving drug regimens, leading to off-label practices for therapeutic and/or economic purposes, and currently, drug regulatory agencies have begun to reappraise this often-neglected topic. This concept also considers the patient’s perspective in terms of quality of life and convenience, including the concept of time toxicity. Overall, the optimization of drug dosing of anticancer agents may be viewed on three sides: the improvement of the benefits/risks balance (patient), the improvement of the convenience of the treatment (patient, healthcare professionals), and the mitigation of the financial impact (health insurance, patient). Examples of dose reassessments of targeted therapies (approved since 1997) are chosen to illustrate the context. Suboptimal/overdosed regimens are found for certain molecularly targeted agents, mostly based on the ancient concept of maximum tolerated dose in oncology. This underlines the lack of comparative effective dose trials before approval. Fortunately, dosing regimens of newly approved molecularly targeted agents is going to evolve with the hope of more convenient and better tolerated treatments. This optimization will bring greater benefit to patients and to healthcare professionals but without addressing the economic issue.

## 1. Introduction

In the past 30 years, major advances in the understanding of cancer biology and drug formulation technologies have led to the development of new classes of anticancer agents based on the identification of pharmacological (actionable) targets in the tumor and in the immune system [1,2]. They refer to molecularly targeted therapies (no official definition) and embrace around 55 molecular alterations. They can be classified into three categories, including (1) biologics such as monoclonal antibodies, antibody–drug conjugates, bispecific antibodies, and fusion proteins; (2) cell therapies such as chimeric antigen receptor (CAR) T cells and tumor-infiltrating lymphocytes; and (3) oral enzyme/protein inhibitors often classified as “small molecules” [3,4,5,6]. Regarding biologics and cell therapies, they have been used since 1997 with rituximab, a monoclonal antibody targeting the antigen CD20 in lymphoma B cells [7], and since 2017 with tisagenlecleucel, a CD19-directed genetically modified autologous CAR T-cell therapy indicated for the treatment of B-cell precursor acute lymphoblastic leukemia (ALL) [8]. Imatinib was the first marketed oral kinase inhibitor (2001) blocking the chimeric deregulated enzyme bcr-abl in chronic myelogenous leukemic cells [9]. Aside from classical anticancer agents (cytotoxic chemotherapy, hormonal agents, radiopharmaceuticals), targeted therapies (including immunotherapies) now dominate the oncology market with around 145 drugs approved as of December 2024. Though designed toward a specific target, agents may be approved for broad tumor indications (without stratification, because the alteration is found in the whole disease) or in a molecularly defined subset of cancer with the need of a companion diagnostic test (called precision medicine).

Although benefiting from a greater understanding of tumor molecular biology and an ever increasing availability of drugs, targeted or biomarker-driven therapy has been criticized mostly because of the oversimplification of the tumoral process and of limited clinical benefits (excepting some rare agents) along with prohibitive prices limiting access (called financial toxicity) [10,11,12]. Overall, the median survival gain brought by targeted agents, including immunotherapy (checkpoint inhibitors), is around 3 months [13,14]. Regarding approved drugs with specific molecular indications (period of 2008–2020), the median gain in overall survival was 4.7 months [15]. Similarly, concerning the period of 2015–2022, substantial clinical benefits evaluated by different scales were only found for 29% of the indications of 50 molecularly targeted agents in the US [16]. In addition, the biomarker-driven treatment of refractory tumors based on an agnostic strategy (i.e., not based on histopathological considerations) proved to be deceptive when compared to conventional therapy [17]. Aside from a moderate or uncertain clinical utility, new anticancer therapeutics are characterized by very high prices impeding the access to patients in various parts of the world. The median cost of an anticancer therapy approved in the US between 2015 and 2020 is around USD 196,000 annually [18]. Overall, and despite a plethora of drugs (in all, around 250 agents), cancer treatment remains characterized by huge needs in therapies with better efficacy and tolerance, particularly in advanced and metastatic disease.

Beyond developing new agents or new combinations of approved agents, cancer treatment can also be optimized by modifying the dosing regimen of approved drugs (dose expression, dosing, dose intensity or dose given per time unit, schedule of administration, and duration of treatment). Academic teams have experimented with different ways to improve drug regimens, leading to off-label practices for therapeutic and/or economic purposes [19,20], and currently, drug regulatory agencies (the FDA or Food and Drug Administration in the US) have begun to reappraise this often-neglected topic [21]. It arises from the rapid development of new molecular targeted agents with sometimes immature or inappropriate dosing regimens. This concept also considers the patient’s perspective in terms of quality of life (toxicity) and convenience, including the concept of time toxicity (time lost by the patient in healthcare facilities in opposition to home time) [22]. In line with the convenience enhancement (less administrations in the hospital setting), the environmental impact of treatment has also begun to be considered (less visits, less drug production, and potential reduced greenhouse gas emissions) [23]. In other words, and paradoxically, new drugs sometimes come to the market with suboptimal dosing regimens, along with uncertainty regarding their therapeutic value (without data on overall survival or quality of life) [24]. In this paper, I shall focus on dosing enhancement strategies for approved anticancer agents. Examples of dosing schedule reassessments for safety concerns of recent approved anticancer targeted agents are presented.

## 2. History of Anticancer Agent Dosing

The first attempt to improve dosing in oncology was the adjustment of the dose in adult patients to body size (weight or estimated surface area) (Table 1). This tradition was based on the works of D. Pinkel at the end of the 1950s, showing that comparable standard doses of rare but existing agents in animals and humans were better obtained when adapted to estimated body surface area than to body weight [25]. It should be mentioned that these works were considering interspecies dose scaling rather than interpatient clinical variability. Consequently, body surface area-based dosing of cytotoxics has been mostly used underscoring the idea that the clearance of drugs is proportional to the size of the elimination organs (liver and kidneys). However, this complicated procedure (calculation of doses with the risk of potential errors) is not really scientifically grounded. Although for some agents, pharmacokinetic parameters have been related to body size in adult patients, and it is unclear whether it translates to significant variations in the clinical response [26]. In the 21st century, body size-based dosing of anticancer agents (as mentioned in the prescription information/official labeling) remains the rule in adult oncology even if some intravenous immunotherapies (nivolumab, pembrolizumab) have moved from a weight-based dosing to a flat dose. This practice is particularly used for intravenous agents because it is easy to perform by adjusting the volume of infusion, and it confers to oncologists an illusive impression of a “precision” dosage. In fact, it has proved to be hypocritical, for example, with monoclonal antibodies such as trastuzumab, rituximab, and daratumumab administered subcutaneously at fixed dose, while the intravenous formulations are used at dosages based on body weight or body surface area according to their prescription information [27,28,29]. Furthermore, and for obvious practical reasons, oral agents are given at flat doses, avoiding the need for multiple strength pills and possible dosing errors for the patient as a result. For example, azacitidine is given orally at a fixed dose and based on body size when injected subcutaneously [30,31].

Later, in the 1970s and 1980s, the relationships between pharmacokinetic variability of anticancer agents and clinical outcomes (efficacy and safety) emerged, leading to the personalized adaptation of dosing based on the determination of drug concentrations in biological fluids (therapeutic drug monitoring) [32,33,34]. Thus, before the era of “precision medicine” and the search for genetic factors of response (or resistance), therapeutic drug monitoring was considered as a major biological tool for improving clinical response in oncology. Regarding the clinical impact (increase of the balance benefits/risks), this approach has been validated for some ancient cytotoxic drugs (methotrexate, etoposide, 5 fluorouracil) in randomized trials that have compared classical body size-based doses to individual pharmacokinetic guided doses [35,36]. In corollary, the benefits of therapeutic drug monitoring proved that dosing based on body size was not sufficient or irrelevant to attenuate pharmacokinetic and clinical interindividual variability. Although mentioned in some official labels (busulfan, methotrexate) or drug regulatory guides, this practice is not universally adopted (excepting the monitoring of methotrexate concentrations after high dose intravenous infusion and the adjustment of folinic acid dosing as a rescue agent), mainly due to beliefs or logistical/practical reasons [36].

Much research has also been conducted to determine the sources of the inter-individual pharmacokinetic/pharmacodynamic variability observed in cancer therapy and in particular the identification of germline variants of genes coding for drug metabolizing enzymes [37]. The main genetic works have focused on the catabolizing enzymes thiopurine methyltransferase or TPMP (6 mercaptopurine), uridine diphosphate glucuronosyltransferases 1A1 or UGT1A1 (irinotecan), dihydropyrimidine dehydrogenase or DPYD (5 fluorouracil, capecitabine) [38]. The identification of particular variants (those conferring a high risk of toxicity due to a low expression of the enzyme) can be performed a priori, before the start of the treatment with the aim to reduce the dosage before the occurrence of severe toxicity. Reducing a priori doses of fluorouracil and irinotecan through dual genotyping of *DPYDA* and *UGT1A1* has proved to significantly reduce severe side effects without impacting survival in patients with gastrointestinal cancer [39]. Again, this approach is conducted in certain specialized centers worldwide, but in France, it is now mandatory for 5-fluorouracil and oral fluoropyrimidines (capecitabine) to be combined with dosages of plasma uracil (endogenous compound) that can identify, as a phenotype test, a severe deficit in DPYD and the need to reduce the starting dose [36]. Interestingly, an anticancer agent (CAR T-cell therapy) has been approved for the first time (November 2024 in the US) with a dosing based on the tumoral burden [40]. Obecabtagene autoleucel (or obe-cel) is a CD19-directed CAR T-cell therapy indicated in the treatment of adults with refractory B-cell precursor acute lymphoblastic leukemia. To mitigate immune side effects, the dose of obe-cel is selected on a bone marrow leukemic blast of >20% or ≤20% [40].

Otherwise, research on variability in drug response has been extending with the ex vivo pharmacotyping of anticancer agents combined with tumoral genotyping in children with acute lymphocytic leukemia [41]. In addition, the impact of the gut microbiome on the pharmacokinetics of some anticancer agents (capecitabine) as a source of variability begins to emerge [42]. Finally, time of administration (circadian variability) may affect the pharmacology and the efficacy of immunotherapies [43,44]. These latter factors of intrinsic pharmacological variability have currently not led to dosing modification in clinical practice.

Currently, excepting pharmacokinetic/germline pharmacogenetics guided dosing, most of the individualization aspects of dosing are performed quite classically, based on the tolerance, the efficacy, the existence of organ dysfunctions (renal/liver insufficiency), the presence of interacting drugs, and the health status/wish of the patient [45,46].

## 3. Post-Approval Dosing Reassessment to Improve the Balance of Benefits/Risks

### 3.1. Approval Dosing and Tolerance Concerns

Historically, the recommended dose of anticancer drugs was set as near the maximal tolerated dose (MTD) [47], that is, producing > grade 3–4 toxicity in one third of tested patients. The MTD is not considered as optimal for targeted agents that display different dose–response (efficacy, toxicity) relationships, with the minimal effective dose far below the MTD [48,49]. Molecularly targeted agents are given chronically (oral formulations), and in case of initial overdosing, they can generate clinical toxic events leading to decreased adherence, dose reduction, interruption or discontinuation of the treatment, decreased efficacy, and wastage [50]. The median cost of wastage for dose reduction or discontinuation is estimated to be USD 1,750 (range of USD 43–27,200) for oral anticancer agents approved in the US between 2020 and 2022 [50]. Regarding FDA-approved drugs between 2002 and 2015, 45% of patients treated with oral agents (mostly kinase inhibitors) needed dose reduction due to toxicity in phase 3 studies [51]. In addition, only 25% of oral agents displayed a recommended therapeutic dose below the MTD, leading to less dose modifications in registration trials (38% versus 50%) [51]. More recently (2021), regarding tyrosine kinase inhibitors approved in the US, discontinuations rates vary between 1% and 39% based on clinical trials data [52]. Further, when considering the real-world practice, 65% of a sample of 11,870 Medicare patients were found to discontinue oral ibrutinib over a median period of 2 years (with 45% of discontinuation in the first year) [53].

#### 3.1.1. Tyrosine Kinase Inhibitors in Chronic Myeloid Leukemia

Patients with chronic myeloid leukemia (CML) are treated throughout life with oral tyrosine kinase inhibitors (imatinib, dasatinib, nilotinib, bosutinib, ponatinib, asciminib). The dosing regimens of certain agents have evolved since their initial approval with the goals of reducing toxicity and bringing more convenience to the patients. For example, dasatinib was initially given at 70 mg twice daily, based on preclinical pharmacodynamic data and its relative rapid elimination (plasma terminal half-life: 3–5 h). Because of toxicity (pleural effusions) and the need for dose reductions or interruptions, an optimization trial was conducted comparing 100 mg once daily to the approved schedule [54]. Dasatinib, given at 100 mg once daily, brought the same activity while reducing the toxicity and its consequences (dose interruption/reduction or discontinuation). This new dosing amount is now recommended in patients with CML (prescription information). Further studies showed that dasatinib was even effective at lower doses (20 mg or 50 mg once daily) [55]. Similarly, a dose–response reassessment (post approval phase 2) has been conducted with ponatinib in line with the emergence of dose-related arterial occlusive events. The optimal regimen in terms of benefit/risk balance was found with a dose reduction of ponatinib (from 45 mg to 15 mg) after achievement of response [56,57]. In addition, the possibility of interruption of treatment in patients with molecular response (nilotinib) has been suggested to enhance quality of life with the preservation of efficacy [58]. Some of these modifications have been included on official labels (ponatinib, nilotinib).

#### 3.1.2. Trastuzumab Deruxtecan

Reassessments of initially approved doses can be performed for a new indication. Hence, a randomized phase 2 comparing two doses (5.4 mg/kg and 6.4 mg/kg once every 3 weeks) of trastuzumab deruxtecan, a monoclonal antibody drug conjugate targeting the human epidermal growth factor receptor 2 (HER2 or ERBB2), has been conducted in 152 patients with HER2-mutant non-small lung cancer (NSCLC). The study showed that the lower dose (5.4 mg/kg) was better tolerated than the initial testing dose (6.4 mg/kg) (32% versus 59% > grade 3 events) with similar efficacy, as assessed by the overall response rate [59]. The lower dosage (5.4 mg/kg) was included in the official labeling for this indication. Initially, following a small phase 1 study (24 patients), the recommended phase 2 doses of trastuzumab deruxtecan were set at 5.4 mg/kg or 6.4 mg/kg [60]. Most indications (HER2-positive/low breast cancer, HER-2 positive cancers) have retained the 5.4 mg/kg schedule, excepting the treatment of HER2-positive gastric cancer (6.4 mg/kg), based on efficacy/tolerance issues but without formal comparison of the two doses [61]. For gastric cancer, the higher dose (6.4 mg/kg) was justified to compensate for a curious tumor type-dependent higher systemic clearance than in breast cancer at 5.4 mg/kg [62]. Thus, HER2-mutant NSCLC was the exception with a randomized dose-finding optimization. The difference between the two doses may appear small (20% difference), but antibody-drug conjugates can be assimilated to molecularly targeted chemotherapy, displaying a narrow therapeutic index when compared to naked monoclonal antibodies.

### 3.2. Refinement of the Dose/Effect Relationship

An approved dosage well above the minimal effective dose, along with toxicity concerns and affordability, prompted the conduction of clinical studies testing lower doses than those initially approved by regulatory agencies.

#### 3.2.1. Nivolumab

Nivolumab is a monoclonal antibody that targets the programmed death 1 (PD-1) inhibitory receptor on T cells, initially approved at 3 mg/kg every 2 weeks before the adoption of the 240 mg flat dose. Nivolumab has been tested in various tumors across a very wide dose ranging (×100) between 0.1 mg/kg and 10 mg/kg without underscoring a dose/effect relationship or a dose/PD-1 receptor occupancy effect [63]. Despite the lack of dose-effect demonstration, the relatively high 3 mg/kg dosing was retained in the initial labeling. Not surprisingly, a very low dose of nivolumab (20 mg instead of the approved 240 mg) was shown to increase median overall survival by 3.4 months when combined with chemotherapy in patients with head and neck cancer in India [64]. Beyond increasing access to patients due to the cheaper cost in resource limited settings, this study confirms a pharmacodynamic impact of nivolumab at a dosing far below the approved one.

#### 3.2.2. Ibrutinib

Ibrutinib is an oral Bruton’s tyrosine kinase (BTK) irreversible inhibitor used in various lymphoproliferative diseases. It was initially (2013) approved at 420 mg daily for the treatment of chronic lymphocytic leukemia (CLL). However, based on the phase 1 results, full occupancy of BTK (a pharmacodynamics proxy evaluated in peripheral blood mononuclear cells) occurred at a dose around 140 mg (2.5 mg/kg) [65]. A pilot study showed comparable biological activity of ibrutinib in 11 patients with CLL receiving a reduced daily dose of 140 mg after a cycle at the standard dose of 420 mg/day [66]. These results, along with those suggesting comparable clinical outcomes in patients with reduced dosing for tolerance or drug interaction considerations, question the selection of the recommended dosage [67]. Some authors have addressed these interrogations on ibrutinib optimal dosage in relation with its cardiotoxicity (also including other BTK inhibitors, such as acalabrutinib and zanubrutinib) [68,69]. However, as of 2024, the approved dosage for the treatment of CLL remains 420 mg daily despite the recommendation of the FDA to test lower dosages [70]. By contrast, in April 2023, accelerated approvals of ibrutinib in mantle cell lymphoma (560 mg daily) and marginal zone lymphoma (420 mg daily) were withdrawn in the US due to negative confirmatory trials and safety concerns [71]. Consequently, in the US, contrary to Europe, the high dosage of ibrutinib (560 mg) has been withdrawn.

#### 3.2.3. Sotorasib

Sotorasib is the first marketed oral covalent inhibitor of the mutated protein KRASG12C, a GTPase acting as an oncoprotein in multiple cancers and considered for a long time as an undruggable target [72]. In 2021, sotorasib received an accelerated approval in the US as a single agent for the treatment of KRAS G12C mutated metastatic NSCLC with a daily dosage of 960 mg [72]. For the first time, the FDA required a post-approval dosing reassessment based on initial uncertainties regarding the optimal dose of sotorasib (possible activity at lower doses, nonlinear pharmacokinetics, and burden of the eight daily 120 mg pills taken) [73]. In fact, data from the FDA clearly showed the absence of a dose exposure relationship with a saturation of oral absorption and a comparable kinetic exposure between 180 mg and 960 mg [72].

Nevertheless, the initial 960 mg dosage was confirmed from a regulatory point of view following the results of a post-marketing phase 2 comparing 960 mg (8 pills) to 240 mg (2 pills) in 209 patients with KRAS G12C mutated NSCLC [74]. The overall response rate was superior for the 960 mg dosage (32.7% versus 24.8% for the 240 mg dosage), but overall survival rates were similar (13 months and 11.7 months, respectively). Not surprisingly, the higher dosage came with an increased grade >3 toxicity (35.6% versus 19.3%) with more gastrointestinal side effects and a higher treatment discontinuation rate (12.5% versus 9.6%) [69]. Therefore, it is difficult to understand why the higher dosage has been maintained, bringing no meaningful clinical benefit, more toxicity (diarrhea, vomiting) due to incomplete absorption, and displaying comparable kinetics to the lowest dosage.

Furthermore, following the accelerated approval, the results of the confirmatory phase 3 trial (CodeBreak 200) comparing sotorasib (960 mg) to docetaxel in advanced pretreated KRAS G12C mutated NSCLC did not show an improvement of the overall survival, leading to another requirement by the FDA to conduct an additional trial [75]. This decision has been highly criticized and debated because, besides the maintenance of the accelerated approval, the possibility of worse survival might exist [75,76,77]. Dosing issues, as well as safety and activity concerns, continue to be addressed in a new indication. Sotorasib is being compared to the standard of care in the CodeBreak300 trial at two dosages (240 and 960 mg) in combination with panitumumab, an anti-epidermal growth factor receptor inhibitor, in patients with refractory metastatic KRAS G12C mutated colorectal cancer. The median progression-free survival (primary endpoint) was slightly superior with the 960 mg dosage (5.6 months versus 3.9 months with 240 mg) but again with similar drug exposures [78].

Sotorasib constitutes a case of a first-in class agent and awaited therapy for one the most prevalent oncogene in cancer but with high uncertainties on the optimal dosage and therapeutic value. Again, it is difficult to anticipate a dose–response relationship with comparable kinetic exposures, which underlines the need to secure the optimal dosing before approval. Currently, sotorasib is approved in the EU at 960 mg daily for KRAS G12C advanced mutated non-small cell lung cancer, but the access has been withdrawn in France following the phase 3 (CodeBreak 200) results [79].

#### 3.2.4. Niraparib

Niraparib is an oral poly (ADP-ribose) polymerase (PARP) inhibitor indicated as maintenance therapy in patients with newly or recurrent epithelial ovarian cancer. The phase 1 showed linear pharmacokinetics between 30 mg and 400 mg without dose-limiting toxicity [80]. Although PARP inhibition exceeded 50% at 80 mg in peripheral blood cells, the phase 2 dose was set at 300 mg daily [80]. However, in the phase 3 evaluation of niraparib versus placebo as maintenance in recurrent ovarian cancer, 73% of the patients required a reduction of the dose to 200 mg or 100 mg for toxicity (primarily thrombocytopenia) [81]. Predictive factors of dose reduction to 200 mg were identified as a low baseline platelet count (<150,000/µL) or a body weight < 77 kg, leading to dosing modification in the subsequent phase 3 trial in the newly diagnosed, as well as in the official labeling [81]. Curiously, when using population pharmacokinetic modeling, body weight has no impact on drug exposure, leading to a mysterious link between hematological toxicity and weight with comparable plasma concentrations [82]. Altogether, initial dosing of niraparib was too high and too toxic because it was based on the maximal tolerated dose and not on the minimum effective dose. A much lower dose might have proved to be as effective and less toxic, but the final analysis of the phase 3 in newly diagnosed patients showed no improvement of the overall survival (vs placebo), leaving uncertainties on the clinical utility of niraparib as maintenance treatment in ovarian cancer [83].

In all, these examples illustrate different scenarios of postapproval dosing reassessments with the reduction of the initial dosing (dasatinib, ponatinib, trastuzumab deruxtecan, niraparib), the withdrawal of the high dosage of ibrutinib in mantle cell lymphoma, the maintenance of the approved initial dosing but with uncertainty concerning the therapeutic benefit (sotorasib) or remaining interrogations regarding the optimal dose (nivolumab, BTK inhibitors in CLL).

### 3.3. The Improvement of the Convenience of the Treatment

At large, the appropriate dosing regimen of an anticancer agent has to bring quality of life and convenience for the patient (also included in the Optimus Project) [21]. It means less time spent in healthcare facilities by reduced hospital administrations, shorter durations of perfusion for parenteral agents, and shorter durations of treatment. Facing tolerance concerns but also «time toxicity», it aims to favor more home days spent by the patients, as opposed to hospital days.

#### 3.3.1. Carfilzomib

Carfilzomib (first approved in 2012) is a protease inhibitor used in the treatment of refractory or relapsed myeloma as a single agent or in combination with other anticancer agents. Carfilzomib has a very complicated dosing schedule with dose escalation, various dosing levels (20, 27, 56, 70 mg/m^2^), and different durations of intravenous perfusion, depending on the indication (prescription information). Since its first approval, the dosing schedule has evolved. Based on preclinical data and the inhibition of proteasome activity (the cellular target), it was initially administered intravenously twice a week (20, 27, or 56 mg/m^2^) on 2 consecutive days, 3 weeks per month [84]. To improve convenience, the once weekly regimen (with highest single dosage of 56 or 70 mg/m^2^) has been tested in various associations. The comparison with the twice weekly regimen (27 or 56 mg/m^2^) showed comparable results, leading to the approval of the once weekly dosing (70 mg/m^2^) for certain combinations (daratumumab, dexamethasone) in the US (2022), and it is a common use in clinical practice [85]. Interestingly, carfilzomib has a short terminal half-life (1 h), but as a covalent irreversible inhibitor, it has a long duration of action. Through modeling, it was shown that 70 mg/m^2^, despite a lower kinetic exposure than 56 mg/m^2^ twice weekly, procures a similar proteasome inhibition, maybe in relation to a higher plasma peak concentration [86]. However, in a trial evaluating weekly carfilzomib (70 mg/m^2^) with lenalidomide and dexamethasone, two fatal adverse events occurred, leading to the test of the 56 mg/m^2^ weekly regimen with this combination [87]. In the phase 3 trial in combination with lenalidomide and dexamethasone, carfilzomib (56 mg/m^2^ once weekly) led to similar efficacy and tolerance as the twice-weekly regimen (27 mg/m^2^) [88].

#### 3.3.2. Monoclonal Antibodies

Monoclonal antibodies and bispecific antibodies are given for long durations of treatment with one to four administrations per month in line with their long terminal half-life (>7 days). To create more convenience, numerous trials have tested extending intervals of administration after approval. Some have proved to procure similar efficacy either with the same dose intensity (keeping the same dose delivered per week on average) or lower. For example, extending dose intervals of the checkpoint inhibitors nivolumab (from 240 mg Q2 weeks to 480 mg Q4 weeks) and pembrolizumab (from 200 mg Q3 weeks to 400 mg Q6 weeks) displayed comparable clinical outcomes in registration trials and clinical practice [89]. Similarly, the manufacturer of cetuximab, an anti-epidermal growth factor receptor (EGFR), reported that the weekly approved 250 mg/m^2^ in 2004 was comparable to 500 mg/m^2^ every 2 weeks (an off-label common practice) [90]. By contrast, for teclistamab, a bispecific antibody used in myeloma, the weekly recommended dose can be switched to every other week in patients with a response [91]. For nivolumab, pembrolizumab, and teclistamab, these less frequent dosing schedules have been included on the official labels. With the enhancement of dosing schedules, less administrations in the hospital setting means less visits and travel, which may reduce the emission of greenhouse gas (environmental impact) [24].

However, this is not the rule in clinical oncology. For example, shortening of administration intervals (from 3 to 2 weeks, keeping the same dose intensity) improves by 12-fold the hematological tolerance (neutropenia) of cabazitaxel (a cytotoxic agent) in patients with prostate cancer, involving more frequent visits to healthcare facilities [92]. This was also observed with the antibody–drug conjugate gemtuzumab ozogamin, where dose fractionation in 3 days showed a better benefit–risk profile in the treatment of acute myelogenous leukemia than the initially approved single injection [93]. Thus, though more convenient, extending the frequency of administration of an anticancer agent may be deleterious.

### 3.4. Duration of Treatment

The duration of treatment of targeted agents is either fixed or undetermined, that is to say, until progression of the disease, it is determined by toxicity or patient wish. Long, undetermined durations of treatment among responders with costly agents addresses financial and adherence issues, particularly in a lifelong curative or maintenance setting [94]. In certain clinical situations (chronic lymphocytic leukemia, relapsed non-Hodgkin lymphoma) with comparable agents, both situations exist (fixed duration or continuous administration), meaning that the treatment duration issue has to be addressed a priori in clinical trials to avoid overtreatment and toxicity. For example, in what regards to bispecific CD20-directed CD3 T-cell engagers in pretreated non-Hodgkin lymphoma, glofitamab is given for a maximum duration of 36 weeks while epcoritamab is administered indefinitely (prescription information). In some cases, duration of treatment has been refined post-approval, such as in the treatment of CML. Hence, the possibility of interruption of treatment with nilotinib in patients with molecular responses has been suggested to enhance quality of life with the preservation of efficacy [58]. Nilotinib, initially approved in 2007 (US), had its label updated in 2017 by the FDA to include treatment discontinuation recommendations for CML patients with sustained molecular responses.

## 4. Discussion

Dosing schedules of targeted therapies may not be optimal at their launch and may increase the risk of toxicity. Post-approval reassessments with less frequent, lower dose regimens with similar activity have proved to be feasible. In this light, and according to the FDA, between 2010 and 2022 in the US, 21 (15%) new drugs (mostly targeted therapies) have necessitated a reevaluation of dosage levels after approval due to safety concerns (post-marketing requirements or PMR) [95]. A similar study found that 17.6% (29/165) of new oncology drugs approved by the FDA (2012–2022) had a dosing variation PMR with the need to evaluate lower or alternative dosages [91]. In addition, the timespan was long to fulfill these dosing requirements (around 5 years) [95,96].

Beyond dosing, it has become clear that the whole regimen (calendar or days of administration/interval of administration, duration of treatment) has to be refined in terms of toxicity and convenience. In the US, the Optimus Project led by the FDA (first published in October 2021 and launched in January 2023) aims to optimize dosing of targeted therapies before approval (and not as post-marketing requirements that occur after a median of 6 years post-approval). One of these dispositions is to compare two potentially effective doses in a randomized phase 2 with the possibility to select the lower one [21,97,98].

Of course, drug agencies have to balance the request for more clinical data regarding optimal dosages with rapid access to patients. Nevertheless, appropriate dosing (without dose modification) ensures better tolerance, long term use, and a better chance of disease control. Patients are willing to get more data on new drugs [99]. This improvement will bring greater benefits to patients but also to healthcare professionals in these times of cancer increased prevalence and staff shortages but without addressing the economic issue (unsurprisingly, the price will be adapted). Additionally, economic considerations or pricing issues are not addressed by regulatory agencies. So, it will remain a problem of socioeconomic inequities (pharmacoequity) and difficulties in terms of affordability and access (even in high-income countries where “administrative toxicity” complicates the dispensation) [100,101]. Besides regulatory dosing questions, this highlights the importance of academic research on dose optimization of targeted therapies that offers the possibility of treatment at a lower cost, as well as a potential for greater tolerance with similar efficacy. When compared with dosing strategies of ancient cytotoxics (chemotherapy), targeted agents mostly benefit from fixed dosing (i.e., not based on body size). In addition, they have not been the subject of pharmacogenetic dosing optimization in clinical practice. Therapeutic drug monitoring of kinase inhibitors is performed in specialized centers, but there is a lack of validation through randomized trials [102].

## 5. Conclusions

These examples illustrate concerns about dosing regimens for certain molecularly targeted agents, partly based on the ancient concept of maximum tolerated dose in oncology (Table 2). This underlines the lack of comparative effective dose trials before approval, translating into a loss of efficiency in drug development. In recent years, several drugs have been the subject of dosing schedule reassessments after approval, sometimes leading to labeling modifications by drug agencies but with delays, leaving other alternatives to off-label use (i.e., under the responsibility of health practitioners), and mainly for economic reasons. Fortunately, the dosing regimens of newly approved molecularly targeted agents are going to evolve with the hope of more convenient and better tolerated treatments.

## Figures and Tables

**Table 1:** Dosing strategies of anticancer agents in adult patients.

Dosing Strategy	Comments
Adaptation to body size (body weight ou estimated body surface aera)	Mostly used for injectable agents (easy to adjust). Assumed to be more precise and to reduce inter-individual variabilty. Not really scientifically demonstrated.
Adaptation to pharmacokinetic parameters (therapeutic drug monitoring)	Used for certain agents; pharmacokinetic variabilty linked to clinical issues
Adaptation to pharmacogenetic characteristics	Used for certain agents; pharmacogenetic characteristics linked to clinical issues (toxicity)
Fixed dose	Used for convenience issues for oral agents or some monoclonal antibodies given subcutaneously or intravenously

**Table 2:** Examples of post approval dosing schedules reassessments of molecularly targeted anticancer agents.

Drug	Modality	Benefits	Labeling Impact
Ibrutinib	Reduced dose in chronic lymphocytic leukemia	Less toxicity	no/off-label
	Withdrawal of high dose in mantle cell lymphoma	No exposition to an unapproved indication	yes (US)
Dasatinib, ponatinib	Reduced dose in chronic myeloid leukemia	Less toxicity	yes
Nilotinib	Treatment interruption in responders with chronic myeloid leukemia	Less toxicity; more convenient; less financial burden	yes (US)
Trastuzumab deruxtecan	Reduced dose in *HER2*-mutated non-small cell lung cancer	Less toxicity	yes
Sotorasib	Initial dose confirmed in *KRAS G12C* non-small cell lung cancer	No demonstrated benefits; potentially mor toxic	yes
Niraparib	Reduced dose in maintenance treatment of ovarian cancer according to the body weight	Less toxicity	yes
Carfilzomib	Reduced frequency of administration in myeloma	More convenient	yes
Nivolumab	Reduced dose in head and neck cancer	Less financial burden	no (off-label)
	Extended frequency of administration	More convenient	yes
Pembrolizumab	Extended frequency of administration	More convenient	yes
Cetuximab	Extended frequency of administration	More convenient	no (off-label)
Teclistamab	Extended frequency of administration in responders with chronic lymphocytic leukemia	More convenient	yes (US)

## Data Availability

Not applicable.
